# Differentiation of Long Non-Coding RNA and mRNA Expression Profiles in Male and Female *Aedes albopictus*

**DOI:** 10.3389/fgene.2019.00975

**Published:** 2019-10-14

**Authors:** Ye Xu, Yunqiao Dong, Yazhou Xu, Zetian Lai, Binbin Jin, Yanqiang Hao, Yonghui Gao, Yan Sun, Xiao-guang Chen, Jinbao Gu

**Affiliations:** ^1^Guangdong Provincial Key Laboratory of Tropical Disease Research, Department of Pathogen Biology, School of Public Health, Southern Medical University, Guangzhou, China; ^2^Reproductive Medical Center of Guangdong Women and Children Hospital, Guangdong Women and Children Hospital, Guangzhou, China; ^3^Department of Laboratory Medicine, Guangdong Women and Children Hospital, Guangzhou, China

**Keywords:** lncRNAs, *Aedes albopictus*, transcriptome, sex, mosquitoes

## Abstract

The Asia tiger mosquito (*Aedes albopictus*) is an important vector of arboviruses, and females can transmit pathogens such as the dengue, zika, and chikungunya viruses. Understanding sex-related differences in this mosquito is fundamental for vector control. However, there are no reports of systematic characterization of long non-coding RNAs (lncRNAs) in male and female *Ae. albopictus*. To investigate the roles of coding and non-coding RNAs in both sexes of *Ae. albopictus*, RNA sequencing was performed on male and female samples. The results showed 305 differentially expressed protein-coding genes (DEGs) between males and females, of which 198 were highly expressed in males and 125 were highly expressed in females. Sex-associated gene ontology terms were enriched. Analysis with the FEELnc software identified 2,623 novel lncRNAs, of which 26 showed male high expression and 11 showed female high expression. Quantitative real-time PCR of randomly selected DEGs and lncRNAs supported the validity of the RNA sequencing results. Knocking down male high-expressed gene AALF000433 in male adults reduced the egg hatching rate. This study provides valuable data on sex-specific expression of protein-coding genes and lncRNAs in adult *Ae. albopictus*, which will guide further studies aimed at understanding sex development and determination mechanisms in this species.

## Introduction

*Aedes albopictus*, an important vector of arboviruses such as dengue (Paupy et al., 2010), zika (Liu et al., 2017), and chikungunya (Vega-Rúa et al., 2014), is an aggressive, invasive mosquito species with a worldwide distribution. Consequently, it is a significant threat to human health. Vector control is a useful way to prevent epidemics of mosquito-borne infectious diseases. Gene drive systems have been proposed as a means of vector control, by either inhibiting the target population or spreading effector genes that make the population refractory to the relevant disease pathogens (Adelman and Tu, 2016). As only the adult female *Ae. albopictus* feeds on animal blood and transmits pathogens, a possible approach to reduce disease transmission is the release of only male *Ae. albopictus*. Hence, it is important to understand the sex determination mechanism(s) used by this species.

In recent years, advances in next-generation sequencing technology and bioinformatic tools have afforded a better understanding of the pathways underlying different biological processes and of the key factors related to such processes, e.g., mRNAs, long non-coding RNAs (lncRNAs), and microRNAs. Access to the genomic sequence from *Ae. albopictus* (Chen et al., 2015; Miller et al., 2018) has also facilitated the development of various omics technologies (Tsujimoto et al., 2017) in this species. More recently, RNA sequencing (RNA-Seq) studies in *Ae. albopictus* have explored differentially expressed mosquito genes in chikungunya virus-infected and uninfected mosquitoes; the transcriptomic changes after dengue virus infection have also been examined (Tsujimoto et al., 2017). Clearly, the use of omics technologies has strong potential to enhance our understanding of the interactions between viruses and their hosts.

lncRNAs form a class of transcripts that are longer than 200 nucleotides but do not encode proteins (Esteller, 2011). Strong evidence is accumulating that lncRNAs have regulatory roles in some major biological processes, including transcriptional and post-transcriptional gene regulation, regulation of genomic stability, and epigenetic regulation. lncRNAs are transcribed from various regions, including promoters and intragenic, intergenic, exonic, and intronic regions, as well as enhancer sequences and antisense strands, based on their genome positions (Jarroux et al., 2017). Most lncRNAs are retained in the nucleus, presumably to target the transcription machinery and for nuclear genome surveillance. lncRNAs have also been reported to be differentially expressed under diverse conditions (Hung et al., 2011). Thus, lncRNAs are currently of great interest in mosquito research and so far, 2,949 putative lncRNAs have been found in *Anopheles gambiae* (Jenkins et al., 2015).

In *Ae. albopictus*, the potential relationships of lncRNAs with sex- or sexual reproduction-associated processes are beginning to attract research attention. Here, we analyzed in detail the mRNA and lncRNA profiles of male and female *Ae. albopictus*.

## Materials and Methods

### Mosquitoes

The *Ae. albopictus* Foshan (Guangdong, China) strain used in this research (established in the laboratory in 1981) was provided by the Center for Disease Control of Guangdong, China. *Ae. albopictus* mosquitoes were reared in an artificial climate box at 25 ± 1°C, with a 10:14 h daily light–dark cycle, and 70–80% relative humidity. Larvae were fed on fish food (Yee^®^, Shandong, China) and the adults were fed on a 10% glucose solution. Three replicons of both male or female *Ae. albopictus* were collected two days after emergence. Each pool contained 15 male or female *Ae. albopictus* individuals.

### RNA Extraction

RNA was extracted from each pool using TRIzol (Invitrogen, Carlsbad, CA, USA), according to the manufacturer’s recommendations. RNA quality was determined using a 2100 Bioanalyzer (Agilent), and the concentration was determined using an ND-2000 instrument (NanoDrop Technologies). Ribosomal RNA (rRNA) was removed from the RNA preparations using an Epicentre Ribo-Zero rRNA Removal Kit (Epicentre, Madison, WI, USA). The rRNA-depleted RNA was used to prepare sequencing libraries with the NEBNext^®^ Ultra™ Directional RNA Library Prep Kit for Illumina^®^ (New England Biolabs, Ipswich, MA, USA), according to the manufacturer’s instructions. The prepared libraries were then sequenced on the Illumina HiSeq™ 4000 platform (2 × 150-bp read lengths) by Gene Denovo Technologies (Guangzhou, China).

### Sequencing Data Analysis

An analysis pipeline was employed to explore the transcriptome profile and identify novel lncRNAs (**Figure S1**). High-quality clean reads were obtained by processing the raw data to remove adapter sequences, low-quality reads, and poly-N sequences using Trimmomatic version 0.35 (Bolger et al., 2014). Next, the clean reads were mapped to the genome of *Ae. albopictus* strain Foshan (Chen et al., 2015; Miller et al., 2018) using Hisat2 version 2.1.1 (Pertea et al., 2016) with the default parameters. StringTie version 1.3.6 (Pertea et al., 2015) was employed to assemble and quantify transcripts with *Ae. albopictus* Foshan strain reference annotation (AaloF1.2, VectorBase, https://www.vectorbase.org) (Lawson et al., 2008). The Ballgown package version 2.16.0 (Frazee et al., 2015) was used to analyze the differentially expressed protein-coding genes (DEGs) in the R (version 3.6) environment (R Core Team, 2013). Fragments per kilobase per million reads was used to measure expression levels of protein-coding genes (Mortazavi et al., 2008). Low-abundance transcripts with a variance across samples of less than one were filtered out. DEGs were defined as genes with two-fold or greater changes between samples from male and female mosquitoes with false discovery rate (FDR) < 0.05. All assembled transcript sequences were generated and reannotated with the eggNOG-mapper (Huerta-Cepas et al., 2015), based on the eggNOG 4.5.1 database (Huerta-Cepas et al., 2015). For enrichment analysis, the R package ClusterProfiler version 3.12.0 (Yu et al., 2012) was used for gene ontology (GO) and Kyoto Encyclopedia of Genes and Genomes (KEGG) pathway enrichment analyses. FDR <0.05 was regarded as the cutoff criterion for both GO and KEGG enrichment analysis.

### Identification of Novel lncRNAs, Data Analysis, and Functional Enrichment Analyses for lncRNAs

*Ae. albopictus* lncRNA loci were detected using FEELnc version 0.1.1 (Wucher et al., 2017), a program that predicts lncRNA loci using a random forest model trained with multi k-mer frequencies and relaxed open reading frames. First, the FEELnc filter module was used to identify non-lncRNA transcripts from reconstructed transcripts of StringTie version 1.3.6, as described in the previous section. The FEELnc filter module also filters out short transcripts (default 200 nucleotides) and can deal with single-exon transcripts. Second, the FEELnc coding potential module (FEELnc codpot) was used to compute a coding potation score for each of the candidate transcripts in the output of the FEELnc filter module. Third, predicted lncRNA transcript sequences were searched against the National Center for Biotechnology Information (NCBI) nonredundant protein database by BLASTX (McGinnis and Madden, 2004). Transcripts with significant homology to known proteins (e.g., e-value < 1e-10, target coverage > 80%, and identity > 90%) were removed. Last, the FEELnc classifier module (FEELnc classifier) was employed to classify *Ae. albopictus* lncRNAs into two major types (“genic” and “intergenic”) and six subtypes. The FEELnc classifier module was used for possible function prediction of differentially expressed lncRNAs based on their nearest-neighbor protein-coding genes. Functional annotation enrichment analyses of differentially expressed lncRNAs were performed as described in the previous section.

### Quantitative Real-Time PCR (qRT-PCR) Validation

Five significantly differentially expressed lncRNAs and mRNAs were randomly selected. Expression levels of selected lncRNAs and mRNAs in the whole body, midgut (MG), and salivary gland (SG) of both males and females, and in the male testis and female ovary were validated by qRT-PCR with three biological replicates. RNA and cDNA were synthesized from RNA isolated from each organism using M-MLV reverse transcriptase (Invitrogen). qRT-PCR was performed using SYBR Green assays with specific primers (**Table S1**), each in a total reaction volume of 20 μl, as described previously (Huang et al., 2016), with a program of 95°C (10 min), 40 cycles of 95°C (10 sec), and 60°C (1 min). β-actin was used for normalization, and the 2^−ΔΔCT^ method (Livak and Schmittgen, 2001) was used to estimate the relative expression of each lncRNA and mRNA. The results were analyzed using student’s *t*-tests (Prescott, 2004), with *P* <0.05 considered to be statistically significant.

### Production of Double-Stranded RNA (dsRNA)

The total RNA of male adult was extracted using TRIzol reagent (Invitrogen, Carlsbad, CA, USA), according to the manufacturer’s protocol. cDNA was synthesized with oligo-d(T) using a SuperScript™ III First-Strand Synthesis System (Invitrogen, Carlsbad, USA) as described in the manufacturer’s protocol. Sense and antisense segments primers (**Table S4**) with T7 promoter were designed to amplify the templates of a 794 bp segment of AALF000433 (**Figure S3**). PCR amplification was performed using PrimeSTAR^®^ HS DNA Polymerase (Takara Bio, Beijing, China) with conditions as follows: 95°C for 5 min, followed by 30 cycles of 30 s 95°C, 30 s at 58°C, and 60 s at 72°C, with a final extension step of 72°C for 10 min. Amplified PCR products were electrophoresed on 1.2% agarose gels and target fragments were extracted using MiniBEST Agarose Gel DNA Extraction Kit Ver.4.0 (Takara Bio, Beijing, China). Sense and antisense RNAs were synthesized *in vitro* using T7 RiboMAX™ Express RNAi System (Promaga, USA) with 1 μg of DNA template in a total volume of 20 μL, respectively. To anneal the RNA strands, mix equal volumes of complementary RNA reactions together and incubate at 70°C for 10 minutes, then slowly cool to room temperature (∼20 minutes) according to the manufacturer’s protocol. Remaining single-strand RNAs and DNA templates in the reactions were digested by RNase and DNase. dsRNAs were purified by phenol chloroform extraction, ethanol precipitated and re-suspended in nuclease free water. A green fluorescent protein (GFP)-derived dsRNA was used as a control.

### Adult Injection, Mosquito Mating, and Egg Collection

Injections of dsRNAs into 2-day-old single reared adult *Ae. albopictus* males were performed under a dissecting microscope. Doses of approximately 30 ng of AALF000433 dsRNAs or *gfp* dsRNAs were injected into the thorax and immediately transferred to small plastic cups (900 ml, 11 cm top diameter) and provided with 10% sucrose solution. The relative expression levels of AALF000433 were quantified by qRT-PCR five days after injection. Gene expression levels were analyzed by the 2^−ΔΔCT^ method (Livak and Schmittgen, 2001). For each treatment described above, three independent mosquitoes were used as biological replicates. Five days after injection, each injected individual male was transferred to a small cup (250 ml, 8 cm top diameter) with one female (one female crossed with one male per cage) for mating for one day. After fully mating, each single female was placed in an oviposition site to lay eggs. The hatching rate was measured. For each treatment described above, ten independent mosquitoes were used as biological replicates. The results were analyzed using student’s *t*-tests (Prescott, 2004), with *P* < 0.05 considered to be statistically significant.

## Results

### Sequencing Data Overview

High-throughput sequencing was performed on an Illumina HiSeq™ 4000 platform with female and male mosquito samples. After adaptor removal and quality trimming, 69,027,078, 83,886,978, and 76,075,782 clean reads from female samples, and 78,871,518, 91,141,086, and 78,543,476 clean reads from male samples were obtained. In addition, nearly 60% of trimmed reads could be mapped to the *Ae. albopictus* Foshan strain genome (**Table 1**).

**Table 1:** Summary of the sequence data.

Sample ID	Accession Number	Total Reads	Trimmed Reads in Pairs	Mapped Trimmed Reads in Pair to Genome	Mapping Rate
Male_Rep1	SRR7990520	79,819,914	79,740,762	47,075,830	59.04%
Male_Rep2	SRR7990523	92,335,944	69,727,610	55,451,229	60.11%
Male_Rep3	SRR7990524	79,513,458	84,886,736	48,640,497	61.23%
Female_Rep1	SRR7990521	69,820,558	92,250,126	43,964,191	63.05%
Female_Rep2	SRR7990522	84,996,982	79,436,046	51,423,008	60.58%
Female_Rep3	SRR7990519	77,063,256	76,986,574	47,431,052	61.61%

Overall transcript expression datasets of samples were used for principal component analysis. The overall transcript profiles of the male and female libraries showed the highest similarity, indicating that genome-wide variance of the biological replicates was negligible, and remarkable differences were observed between the sexes (**Figure S2**).

### Gene Expression Analysis and DEGs Identification

**Figure 1:**
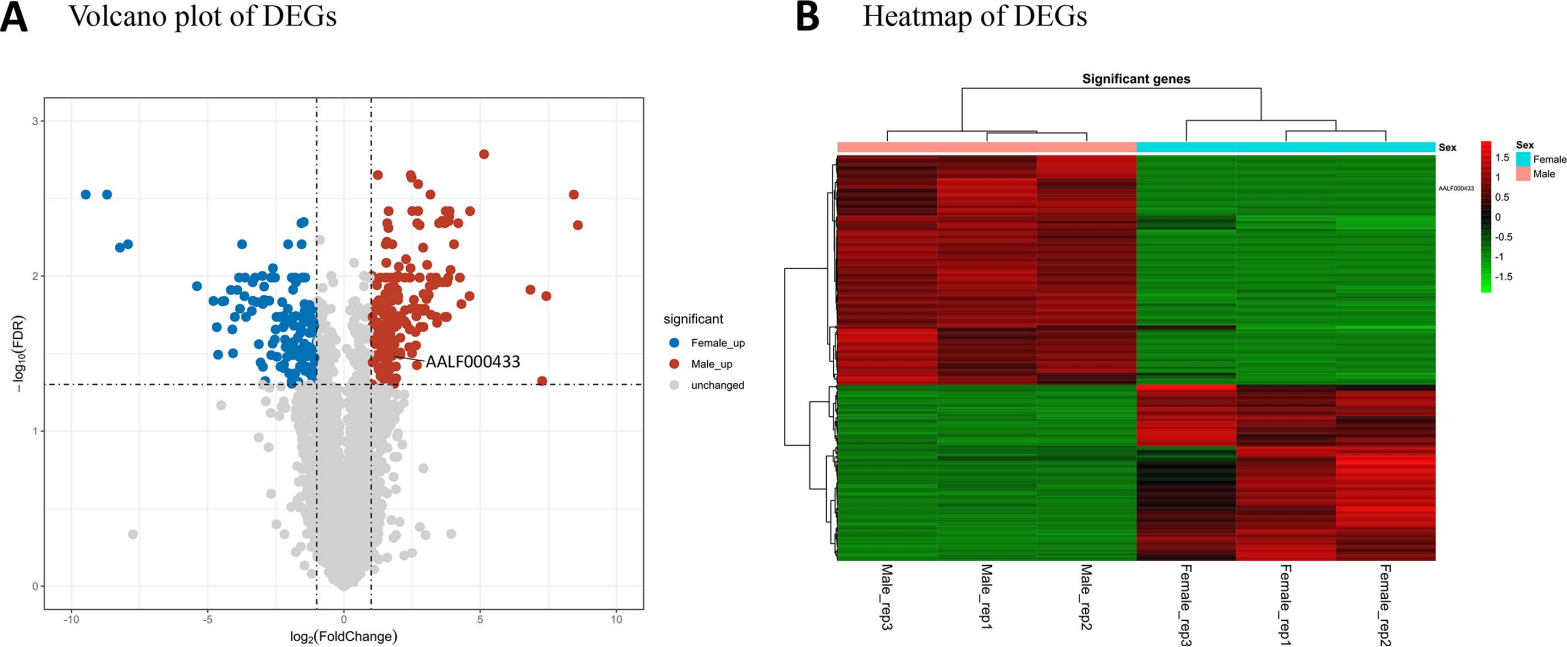
mRNA expression profiles of male and female *Ae. albopictus adults*. **(A)** Volcano plot analysis of gene expression variation. Red and blue points correspond to 2.0-fold changes in male and female high-expression mRNAs, respectively, and indicate FDR  < 0.05. **(B)** Hierarchical clustering of the 305 DEGs in the six samples. Green represents low expression levels and red represents high expression levels.

StringTie version 1.3.6 was used for transcript assembly and detection of expression. A total of 39,492 transcripts from 29,862 protein-coding genes were assembled. A total of 305 protein-coding genes were considered to be differentially expressed between the male and female mosquito samples (**Table S2**). Among these DEGs, 198 had male high-expression and 125 female high-expression. Volcano plot analysis (**Figure 1A**) was used to depict the gene expression distribution. Hierarchical clustering analysis (**Figure 1B**) of 305 DEGs was performed between the different libraries; in the figure, red and green lines indicate male high-expression and female high-expression levels for each library.

### Functional Enrichment of DEGs

To further elucidate the functional roles of the DEGs, we used the eggNOG database and ClustalProfile R package for GO and KEGG pathway enrichment analysis. The top 15 significantly enriched (FDR < 0.05) GO terms in the categories of biological process (BP), cellular component (CC), and molecular function (MF) were obtained.

**Figure 2:**
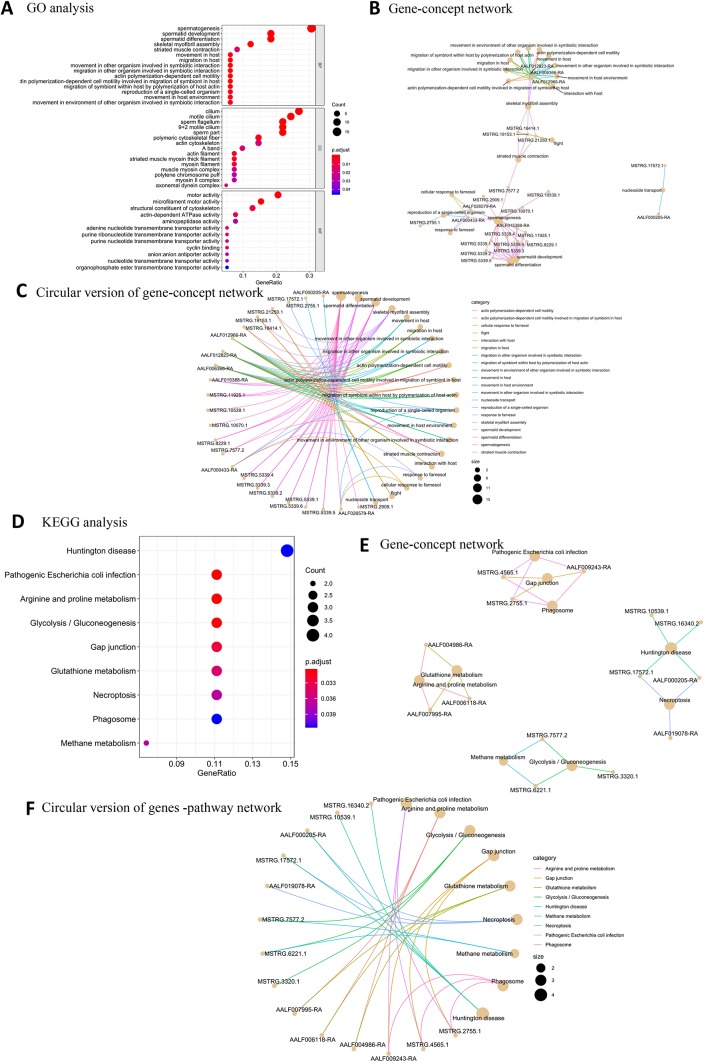
GO and KEGG pathway enrichment analysis of male high-expression protein-coding genes. **(A)** The top 20 GO terms for BP, CC, and MF for male high-expression protein-coding genes. **(B)** Gene-concept network depicting the linkages of male high-expression protein-coding genes and biological concepts as a network. **(C)** Circular version of gene-concept network for male high-expression protein-coding genes. **(D)** Top KEGG pathway terms for male high-expression protein-coding genes. **(E)** Protein-coding gene-pathway network depicting the linkages of male high-expression protein-coding genes and KEGG pathways as a network. **(F)** Circular version of male high-expression protein-coding gene-pathway network.

Among the BP terms for male high-expression protein-coding genes, the most significantly enriched GO terms were related to male sex function (**Figure 2A**): spermatogenesis, spermatid development, spermatid differentiation, and skeletal myofibril assembly (movement in host). The most enriched GO CC terms of male samples were sperm flagellum, 9 + 2 motile cilium, motile cilium, cilium, and sperm part. The MF terms for the male samples indicated that motor activity was very important; representative terms were microfilament motor activity, motor activity, structural constituent of cytoskeleton, actin-dependent ATPase activity, and adenine nucleotide transmembrane transporter activity. Fifteen protein-coding genes, including 13 novel and two known protein-coding genes, were associated with sperm-related terms. (**Figures 2B, C**). KEGG pathway analysis showed that nine overrepresented pathways with FDR of <0.05 were enriched in male samples (**Figures 2D–F**).

**Figure 3:**
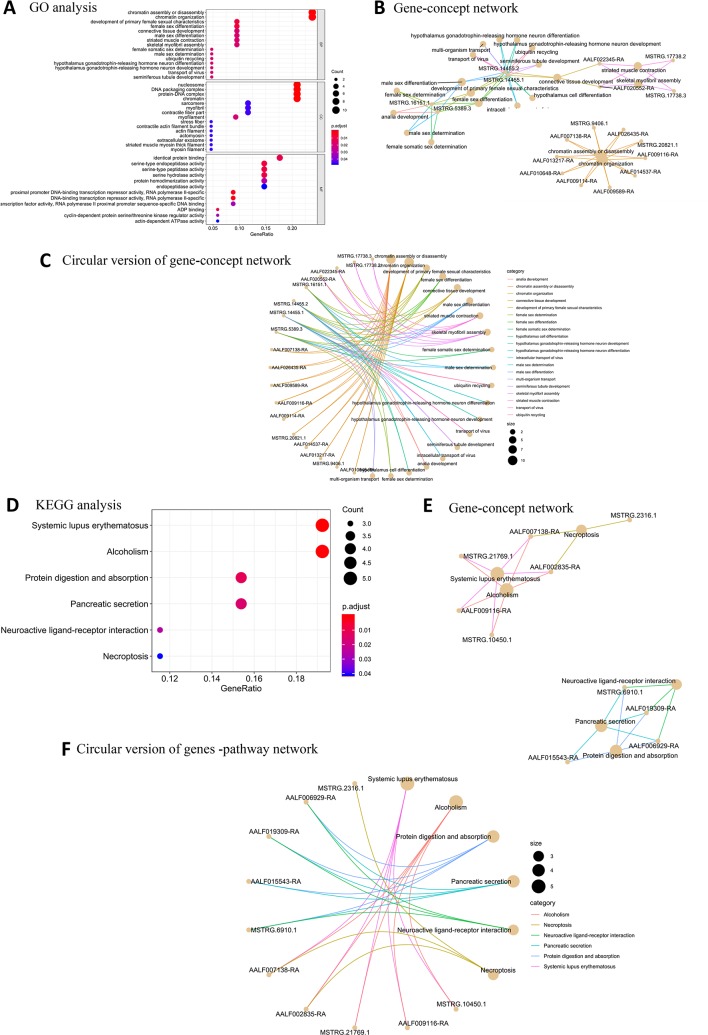
GO and KEGG pathway enrichment analysis of female high-expression protein-coding genes. **(A)** Top 20 GO terms for BP, CC, and MF for female high-expression protein-coding genes. **(B)** Gene-concept network depicting the linkages of female high-expression protein-coding genes and biological concepts as a network. **(C)** Circular version of female high-expression protein-coding gene-concept network. **(D)** Top KEGG pathway terms for female high-expression protein-coding genes. **(E)** Protein-coding gene-pathway network depicting the linkages of female high-expression protein-coding genes and KEGG pathways as a network. **(F)** Circular version of female high-expression protein-coding gene-pathway network.

Among the BP terms for female high-expression protein-coding genes, the most significantly enriched GO terms were related to chromatin assembly or disassembly, chromatin organization, development of primary female sexual characteristics, female sex differentiation, and connective tissue development. The most enriched CC terms were nucleosome, DNA packaging complex, protein-DNA complex, chromatin, and myofilament. The significantly enriched MF terms were proximal promoter DNA-binding transcription repressor activity, RNA polymerase II-specific, serine-type endopeptidase activity, serine-type peptidase activity, and serine hydrolase activity (**Figure 3A**). Four new protein-coding genes (MSTRG.14455.2, MSTRG.14455.2, MSTRG.16151.1, and MSTRG.5389.3) were associated with sex determination (**Figures 3B, C**). Six overrepresented KEGG pathway terms were enriched in the female samples (**Figures 3D–F**).

**Figure 4:**
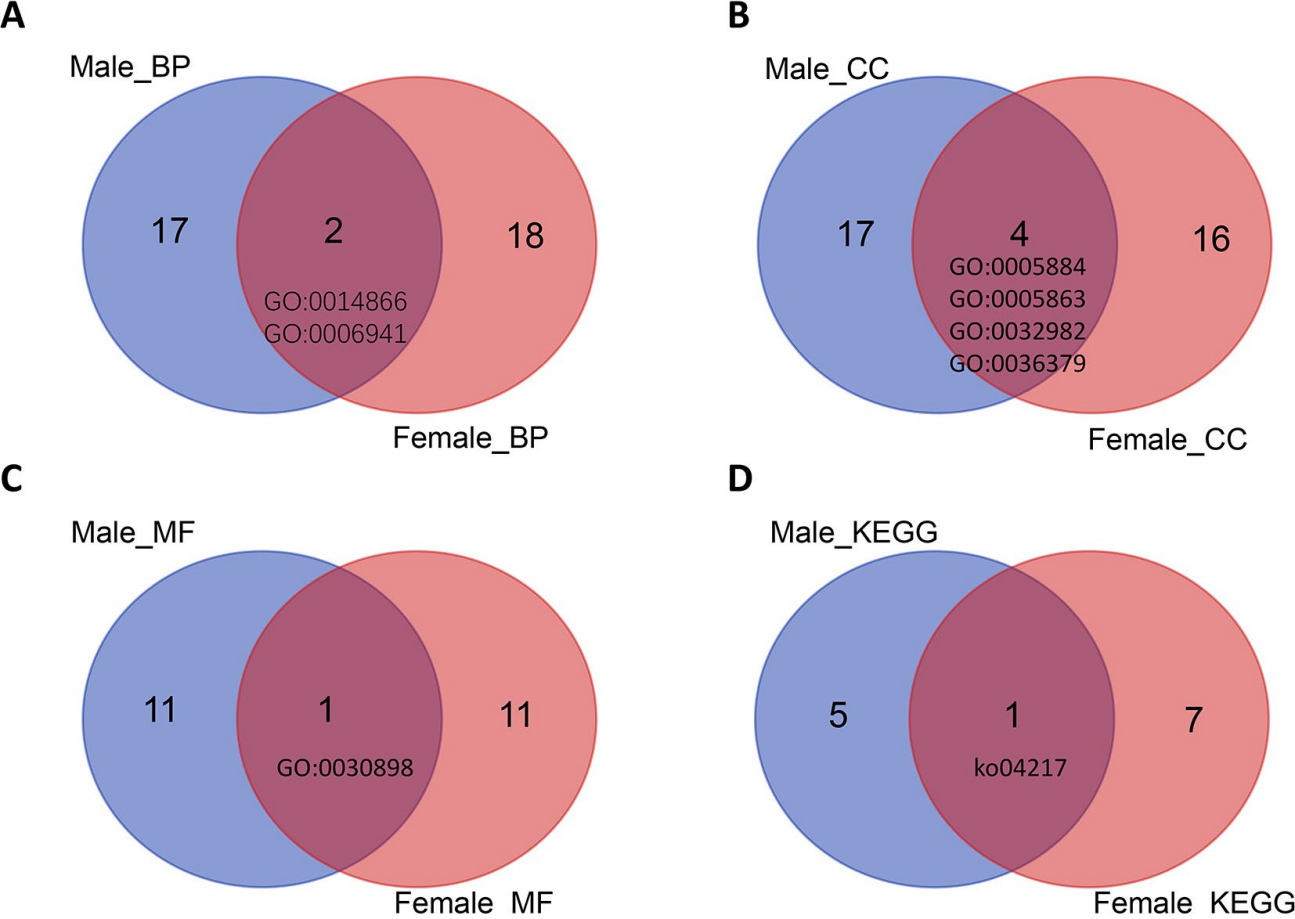
Venn plots of GO terms and KEGG pathway terms for both male and female high-expression protein-coding genes. **(A)** Two BP term was enriched in male and female samples. **(B)** Four CC terms were enriched in male and female samples. **(C)** One MF term was enriched in male and female samples. **(D)** One KEGG term was enriched in male and female samples.

The GO terms skeletal myofibril assembly (**Figure 4A**), actin filament, striated muscle myosin thick filament, myosin filament, myofilament (**Figure 4B**), and actin-dependent ATPase activity (**Figure 4C**) were enriched in both male and female samples with different protein-coding genes. The glutathione metabolism pathway was enriched in both male (AALF004986-RA, AALF006118-RA, AALF007995-RA) and female samples (AALF019309-RA, AALF006929-RA) with different protein-coding genes (**Figure 4D**).

### *Ae. albopictus* lncRNAs Predicted by FEELnc

**Figure 5:**
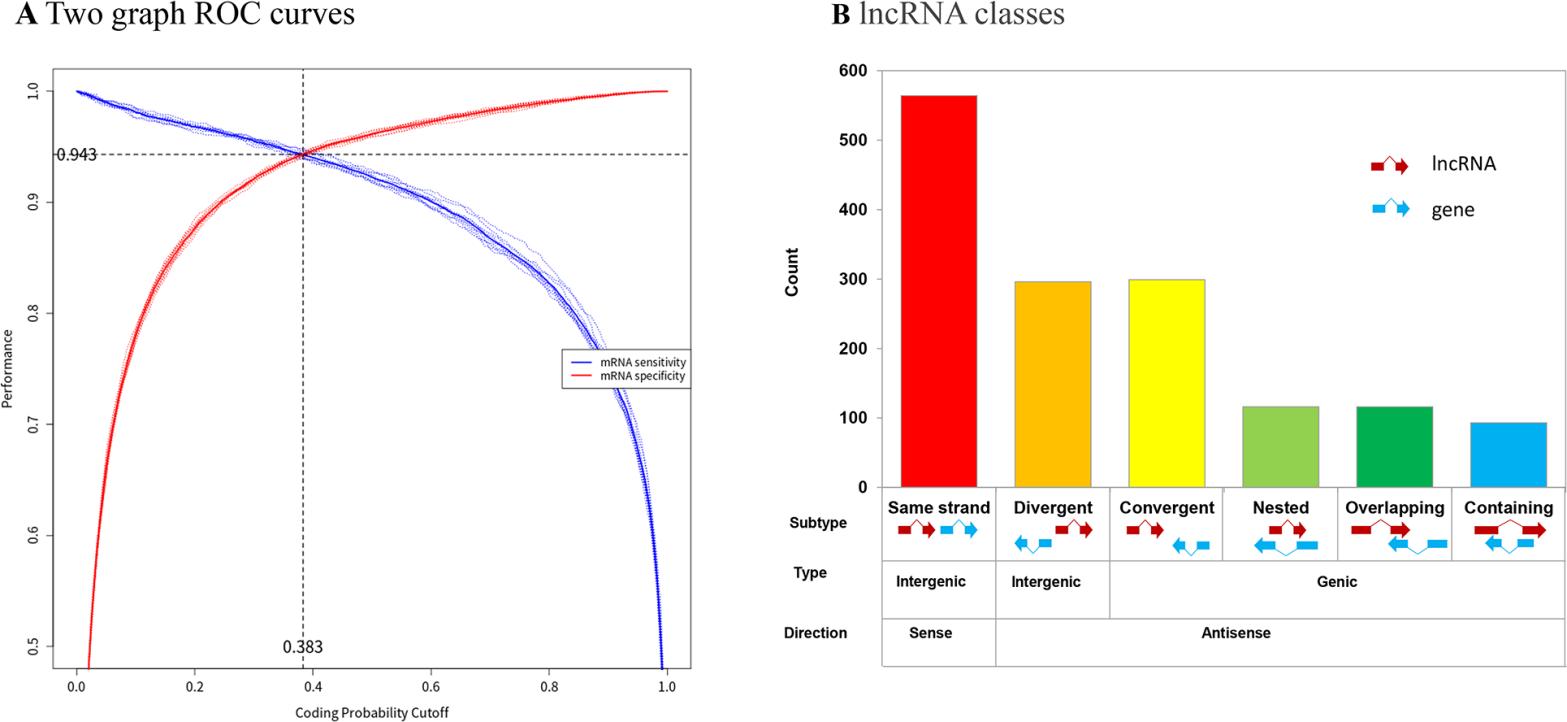
Identification of novel lncRNAs in *Ae. albopictus*. **(A)** Two ROC curves for automatic detection of optimized CPS threshold and user specificity threshold. Red line indicates mRNA specificity. Blue line indicates lncRNA specificity. **(B)** lncRNA classes based on RNA partners from reference annotations. Types and counts of novel lncRNA molecules in *Ae. albopictus*.

The optimal coding potential score (CPS) was used to determine the coding status of the RNA gene models. FEELnc automatically computes the CPS cut-off that maximizes classification performance and provides users with a two-graph receiver operating characteristic (ROC) curve to display the performance of the model and visualize the CPS. The results showed a CPS cut-off of 0.383 (**Figure 5A**). In total, 2,623 novel lncRNAs were identified. Using the FEELclassifier module, the class distributions of the 1484 lncRNAs were classified by comparison with annotated protein-coding genes from VectorBase. We found 325 genic and 1,159 intergenic lncRNAs (**Figure 5B**).

### Identification of Differentially Expressed lncRNAs Between Females and Males

**Figure 6:**
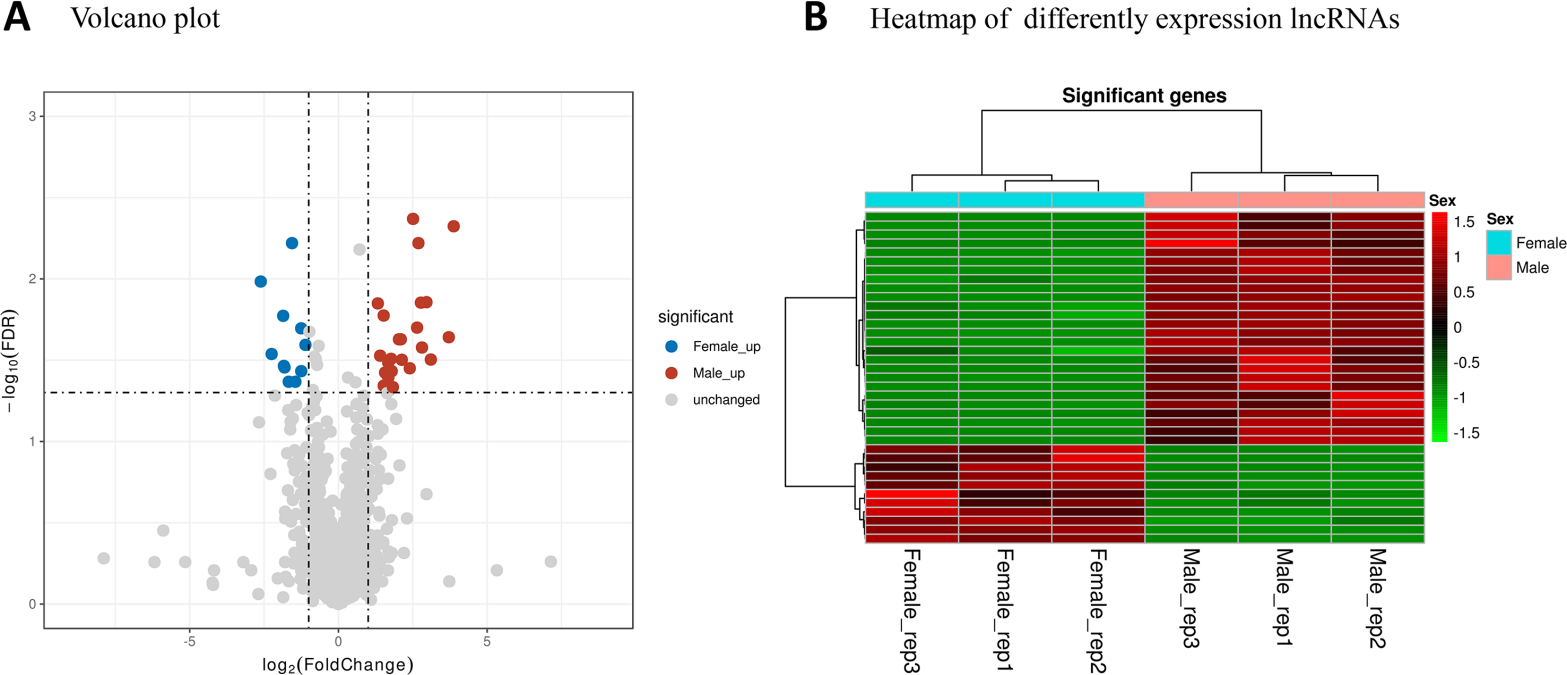
lncRNA expression profiles in male and female *Ae. albopictus* mosquitoes. **(A)** Volcano plot analysis of lncRNA expression variation between males and females. Red and blue points correspond to 2.0-fold changes in male/female high-expression mRNAs, respectively, and indicate FDR  < 0.05. **(B)** Hierarchical clustering of the differentially expressed lncRNAs. Green represents low expression levels and red represents high expression levels.

Differentially expressed lncRNAs between the male and female samples were also analyzed. A total of 37 lncRNAs were considered to be differentially transcribed between the male and female samples (**Table S3**). Among these, 26 male high-expression lncRNAs and 11 female high-expression lncRNAs were detected (**Figure 6**).

**Figure 7:**
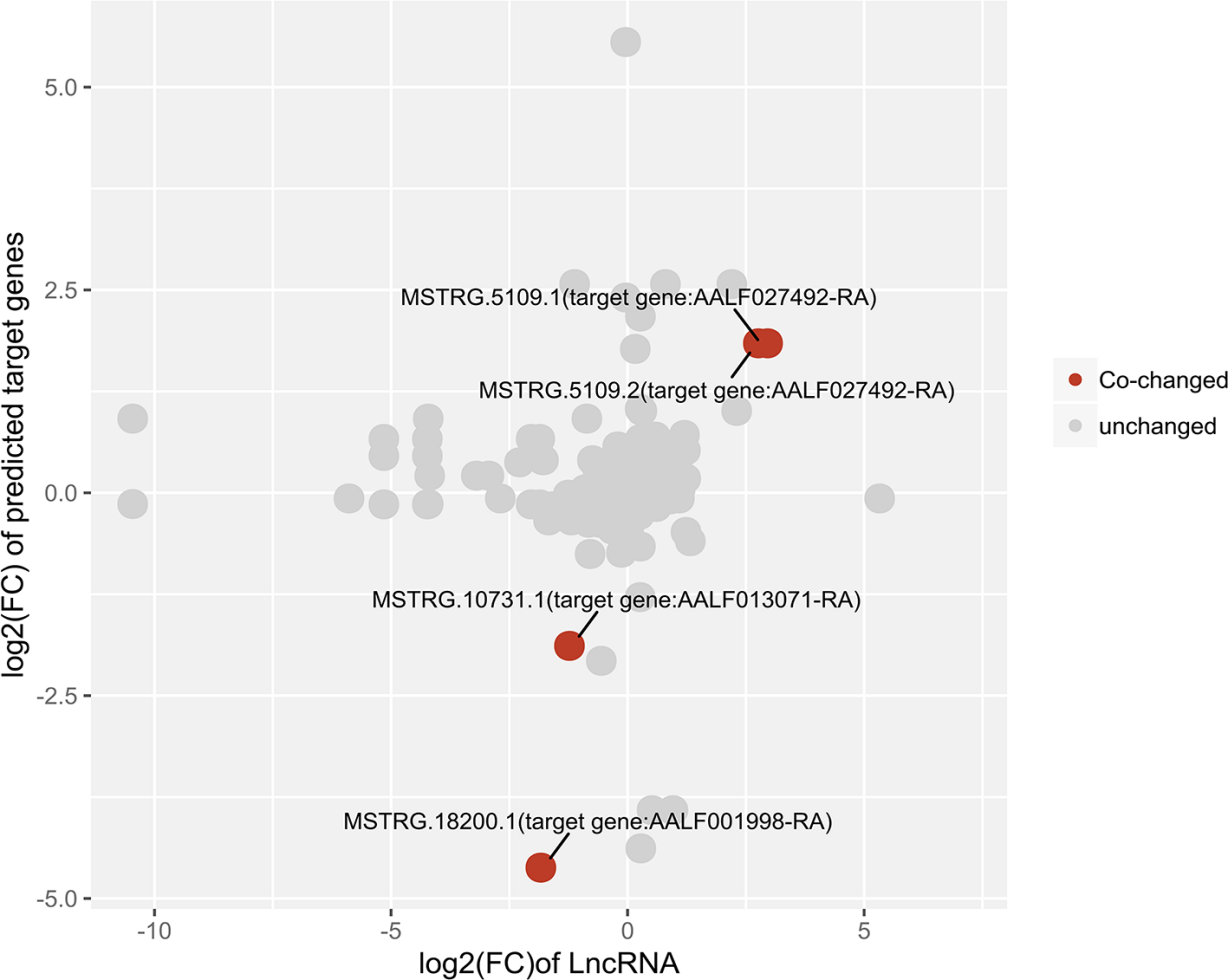
Correlation plot of lncRNA:mRNA pairs of differentially expressed lncRNAs and their predicted target protein-coding genes.

The expression relationships between differentially expressed lncRNAs and their target protein-coding genes were also analyzed. The results showed co-high expression of two significant male high-expression lncRNAs and their target genes using relaxed criteria (*P* <0.05 for both lncRNAs and protein-coding genes). Two female high-expression lncRNAs showed co-high-expression with their target genes (**Figure 7**).

### GO and KEGG Pathway Analyses of Protein-Coding Genes Targeted by Differentially Expressed lncRNAs

**Figure 8:**
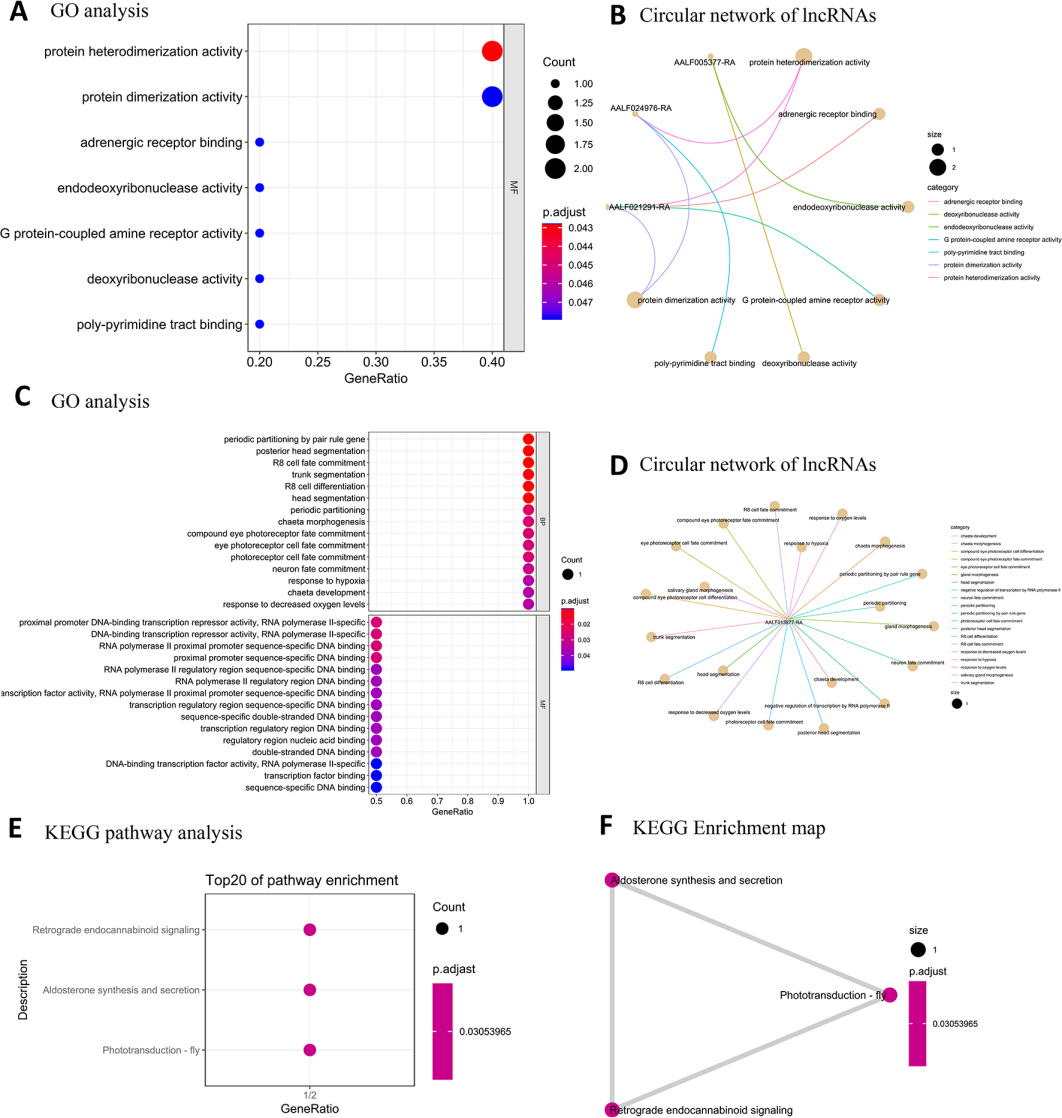
GO and KEGG pathway enrichment analysis of male/female high-expression lncRNA target protein-coding genes. **(A)** Top GO terms for MF for male high-expression lncRNA target protein-coding genes. **(B)** Circular network of male high-expression lncRNA target protein-coding genes and BCs. **(C)** Top GO terms for female high-expression lncRNA target protein-coding genes. **(D)** Circular network of female high-expression lncRNA target protein-coding genes and BCs **(E)** Top KEGG pathway terms for female high-expression lncRNA expression. **(F)** Enrichment map organizing enriched terms into a network with edges weighted by the ratio of overlapping gene sets.

As lncRNAs are known to regulate their nearest-neighbor protein-coding genes (Rinn et al., 2007; Faghihi et al., 2008; Guttman et al., 2009; Villegas et al., 2014), we performed GO term and KEGG pathway enrichment analysis on nearest-neighbor protein-coding genes to predict the potential function of the differentially expressed lncRNAs. In male samples, seven GO terms of MF were significantly enriched (FDR < 0.05) (**Figures 8A, B**). No overrepresented KEGG pathway was enriched in the male samples. In the female samples, 30 GO terms were significantly enriched (FDR < 0.05) (**Figures 8C, D**). The KEGG pathway analysis revealed that the glutathione metabolism [ko00480] pathway was enriched in the female samples (**Figures 8E, F**).

### Validation of Differentially Expressed lncRNAs and mRNAs

**Figure 9:**
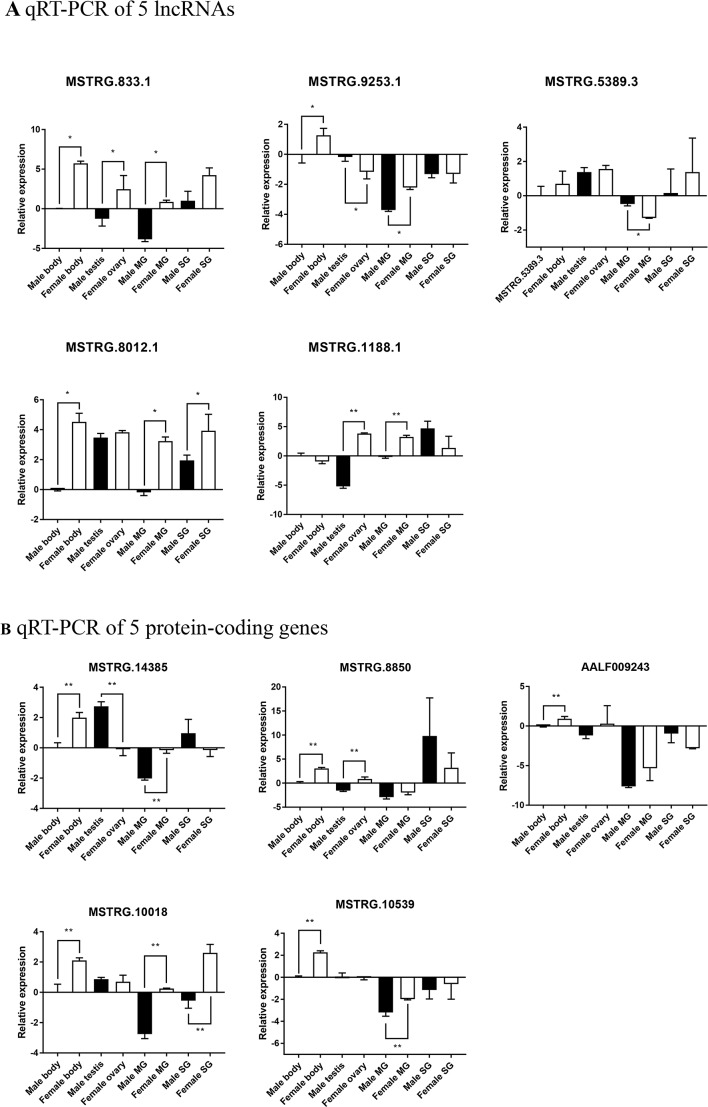
qRT-PCR-based quantitation of male and female lncRNAs and protein-coding genes with significant differential expression levels in whole body, sex organs, MG, and SG. Relative expression levels of **(A)** five lncRNAs and **(B)** five protein-coding genes. Data represent mean ± standard deviation, *P < 0.05, **P < 0.01.

Five significantly differentially expressed lncRNAs and mRNAs were randomly selected and validated using qRT-PCR. According to the results, three selected lncRNAs (MSTRG.883.1, MSTRG.9253.1, and MSTRG.8012.1) (**Figure 9A**) and all five selected protein-coding genes (MSTRG.14385, MSTRG8850, AALF009243, MSTRG.10018, and MSTRG.10539) (**Figure 9B**) showed significant low-expression in male adults, consistent with the RNA-Seq data. These lncRNAs were also detected in the mosquito sex organs, MG, and SG. Three selected lncRNAs (MSTRG.883.1, MSTRG.9253.1, and MSTRG.1188.1) and two selected protein-coding genes (MSTRG.14385 and MSTRG.10539) were significantly differentially expressed between male testis and female ovary. Five selected lncRNAs (MSTRG.883.1, MSTRG.9253.1, MSTRG.5389.3, MSTRG.8012.1, and MSTRG.1188.1) and three selected protein-coding genes (MSTRG.14385, MSTRG.10018, and MSTRG.10539) were significantly differentially expressed between male MG and female MG. One selected lncRNA (MSTRG. 8012.1) and one selected protein-coding gene (MSTRG.10018) were significantly differentially expressed between male SG and female SG. Therefore, the qRT-PCR results support the validity of the RNA-Seq results, indicating that these lncRNAs and mRNAs may be involved in sex differences in *Aedes* mosquitoes.

### Knocking Down AALF000433 in Male Adults Could Reduce Egg Hatching Rate

**Figure 10:**
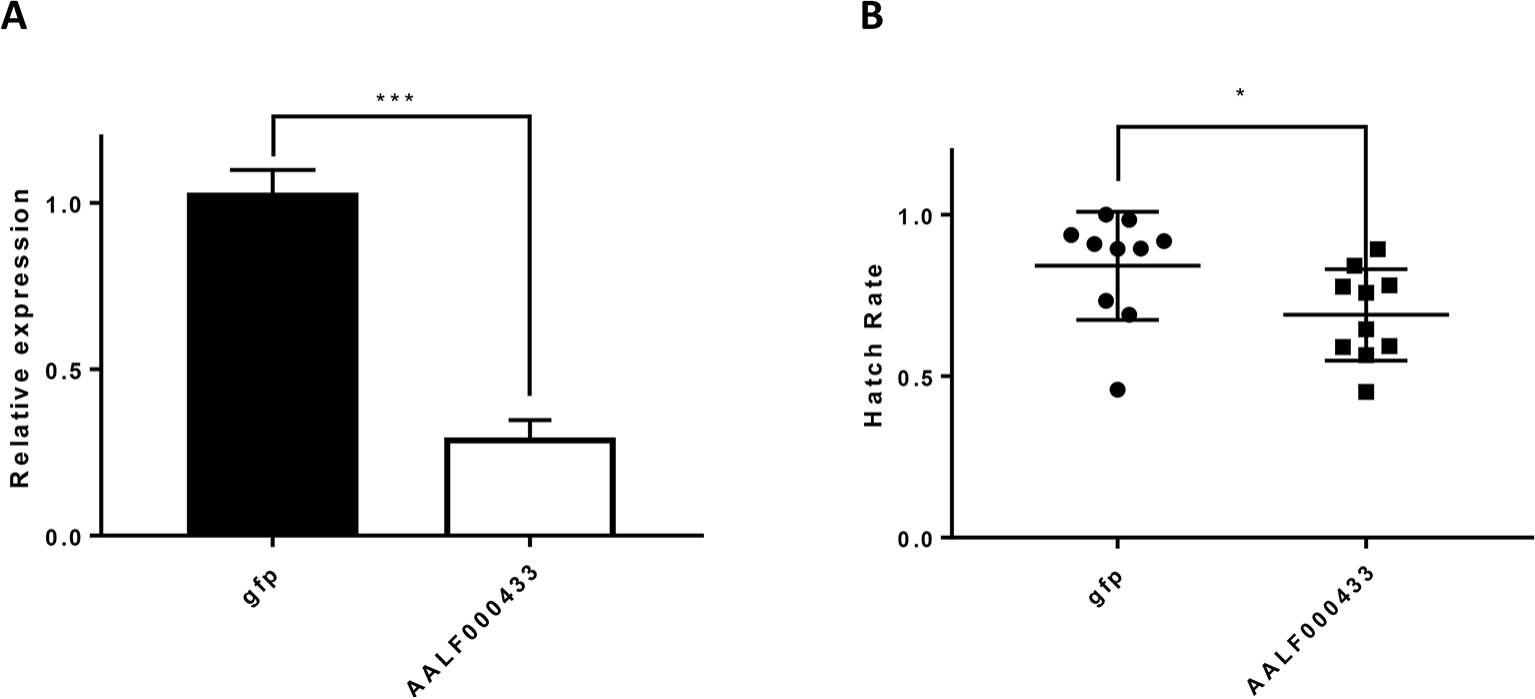
Knockdown of AALF000433 in male adults. **(A)** Analysis of the effects of AALF000433 interference in male adults by qRT-PCR. Bars represent the standard error of the mean (SEM) (n  =  3). The *x*-axis indicates the groups. **(B)** The hatch rate of eggs from each female mate in each treatment group. Data are shown as mean ± SEM (*P < 0.05, ***P < 0.001).

As mentioned in GO enrichment analysis, a male up-regulated gene, the AALF00043 gene, was enriched in the GO category of cellular response to farnesol, reproduction of a single-celled organism, and spermatogenesis (**Figure 2B**). Thus, it was selected for knockdown experiments to verify its association with reproductive activity in *Ae. albopictus*. The gene expression level of AALF00043 was reduced to 29.5% of the normal level five days after injection (**Figure 10A**). Treated male adults were mated with single reared females individually. Hatching rates of single females were measured. The hatching rate after treatment (62.1 ± 14%, n = 10) was significantly lower than that of untreated controls (84.2 ± 16%, n = 10) (*P* <0.05) (**Figure 10B**).

## Discussion

Various sex determination mechanisms are present in insects. The mechanisms of sex determination in mosquitoes are of particular interest, as only the adult females can bite and transmit diseases. For the past three decades, *Ae. albopictus* has been monitored as the vector for dengue transmission in south China (Jing et al., 2012). With the aim of obtaining further insight into the sex determination mechanisms and sexual reproduction processes in *Ae. albopictus*, we conducted a transcriptomic analysis of coding and non-coding gene changes occurring in males and females of this species. This is the first study to provide data on sex-specific protein-coding genes and lncRNAs expression in adult *Ae. albopictus*.

lncRNAs have regulatory roles in various important biological processes. lncRNA profiles have been studied in several mosquito species (Padron et al., 2014; Jenkins et al., 2015; Etebari et al., 2016), showing associations of lncRNA function with aspects of reproduction such as sex determination (Zhang et al., 2010; Mulvey et al., 2014), meiosis (Shichino et al., 2014), and spermatogenesis (Arun et al., 2012; Nolasco et al., 2012). In 2014, Mulvery et al. showed that transgenic lines ectopically expressing certain lncRNAs could affect the process of *Drosophila* sex determination and alter levels of other lncRNAs (Mulvey et al., 2014). Our study identified 37 lncRNAs that were differentially transcribed between male and female samples; these are promising candidates for exploring the function of lncRNAs in sex determination and development.

FEELnc, an alignment-free program that accurately annotates lncRNAs based on a random forest model trained with general features, and BLASTX (McGinnis and Madden, 2004) were used to identify and classify lncRNAs in this study. FEELnc (Wucher et al., 2017) has been shown to achieve similar or better classification performance on GENCODE (Harrow et al., 2012) and NONCODE (Bu et al., 2011) data sets when compared with five programs [PhyloCSF (Lin et al., 2011), CPC (Kong et al., 2007), CPAT (Wang et al., 2013), PLEK (Li et al., 2014), and CNCI (Sun et al., 2013)], and has previously been used to identify lncRNAs (Che et al., 2018; Donato et al., 2018; Wang et al., 2018). In this study, 2,623 novel lncRNAs were identified, of which 1,484 were classified. Various studies have suggested that lncRNAs can regulate the expression of neighboring genes in *cis* (Rinn et al., 2007; Faghihi et al., 2008; Guttman et al., 2009; Villegas et al., 2014).

The *Ae. albopictus* Foshan genome (Chen et al., 2015) and the corresponding protein-coding gene set annotations facilitate assembly and quantification of transcripts for further lncRNA identification. Using Hisat2-StringTie-Ballgown pipeline (Pertea et al., 2016), 305 DEGs and 37 differentially expressed lncRNAs between male and female samples were identified (|log_2_(fold change)| > 1, FDR < 0.05). As expected, several sperm development BP terms were enriched. Importantly, MSTRG.7577.2, MSTRG.19153.1, MSTRG.18414.1, and MSTRG.21250.1 putatively connect the biological processes of spermatogenesis and cell movement. These DEGs could play important parts in the reproductive development of male sperm. In this study, male high-expression gene AALF000433 was knocked down in male adults using dsRNA. The results showed a strong relationship between knockdown of AALF000433 and a decreased egg hatching rate. The BLAST results showed that AALF000433 encodes the nucleosome assembly protein (Nap1). In 2013, Kimura et al. showed that *Drosophila* Nap1 is localized at the apical tip of the sperm head, and the *nap1* mutant exhibits disruption of the nuclear bundle (Kimura, 2013). This implies that knocking down the expression of *nap1* in *Ae. albopictus* might affect sperm activity, resulting in the low egg hatching rate. Sex determination BP terms were also enriched in female samples (Doublesex [*dsx*] gene isoform, MSTRG.16151.1, and MSTRG.5389.3). The *dsx* gene could have an ultimate discriminatory role for sex determination in insects. The *dsx* pre-messenger RNA produces male- and female-specific splicing patterns that encode the male-DSXM and female-DSXF proteins. These proteins determine male and female development, respectively. The function of *dsx* has been explored in several insects, including *Drosophila* (Shirangi et al., 2016), beetles (Ledón-Rettig et al., 2017), *Bombyx mori* (Xu et al., 2017), *A. gambiae* (Kyrou et al., 2018), and honey bees (McAfee et al., 2019). Previous studies have suggested that lncRNAs can regulate neighboring coding genes (Villegas and Zaphiropoulos, 2015). In the current study, the functions of differentially expressed lncRNAs were predicted by exploring their partner protein-coding genes. Enriched terms indicated that potential target protein-coding genes were associated with DNA-binding molecular functions.

The accuracy of the RNA-Seq analysis was verified by qRT-PCR using five randomly selected significantly expressed protein-coding genes and lncRNAs. All of the results were consistent with the RNA-Seq data, supporting the reliability of the RNA-Seq data. The present research findings expand our current knowledge about lncRNAs and their relationships with sex development protein-coding genes in *Ae. albopictus*.

## Conclusion

We have presented the first genome-wide analysis of lncRNAs from adult male and female *Ae. albopictus*, and identified the specifically expressed lncRNAs and mRNAs in males and females. These results will be of use in further studies to obtain a deeper understanding of *Ae. albopictus* sex determination mechanisms.

## Data Availability Statement

Sequencing data were deposited in the NCBI Sequence Read Archive under the accession numbers SRR7990520, SRR7990523, SRR7990524, SRR7990521, SRR7990522, and SRR7990519.

## Author Contributions

JG and YX conceived, designed, and supervised this study. YX performed data analysis and wrote the first draft. BJ prepared the samples. YD, ZL, YH, YG, YS, and X-g C participated in discussions and provided valuable advice and practical contributions. YX, and YZX participated in knockdown experiment. All authors reviewed, edited, and approved the manuscript.

## Funding

This work was supported by the National Natural Science Foundation of China (81672054), the Natural Science Foundation of Guangdong Province (2017A030313120), the Research Team Program of the Natural Science Foundation of Guangdong (2014A030312016), and the China Postdoctoral Science Foundation (2018M633078).

## Conflict of Interest

The authors declare that this research was conducted in the absence of any commercial or financial relationships that could be construed as a potential conflict of interest.

## Supplementary Material

Figure S1 Overview of transcriptome analysis and lncRNA identification.

Figure S2 Principal component analysis (PCA) of the general transcriptome characteristics. The first principal component (PC1) accounted for 62.3% and the second principal component (PC2) for 23% of the total variance in the dataset. The plot indicates that the transcriptome data are highly reproducible.

Figure S3 Location of dsRNA in AALF000433 and the sequence. Light Blue indicate the sequence of dsRNA.

Table S1 Primers used for qRT-PCR.

Table S2 The detail information of all DEGs.

Table S3 Detailed information for all differentially expressed lncRNAs.

Table S4 Primers for AALF000433 dsRNA and qRT-PCR.
