# Supplementary figures and images for: Differentiation of Long Non-Coding RNA and mRNA Expression Profiles in Male and Female Aedes albopictus
Source: Front Genet. 2019 Oct 14;10:975. doi: 10.3389/fgene.2019.00975 (PMC6802003; doi:10.3389/fgene.2019.00975)

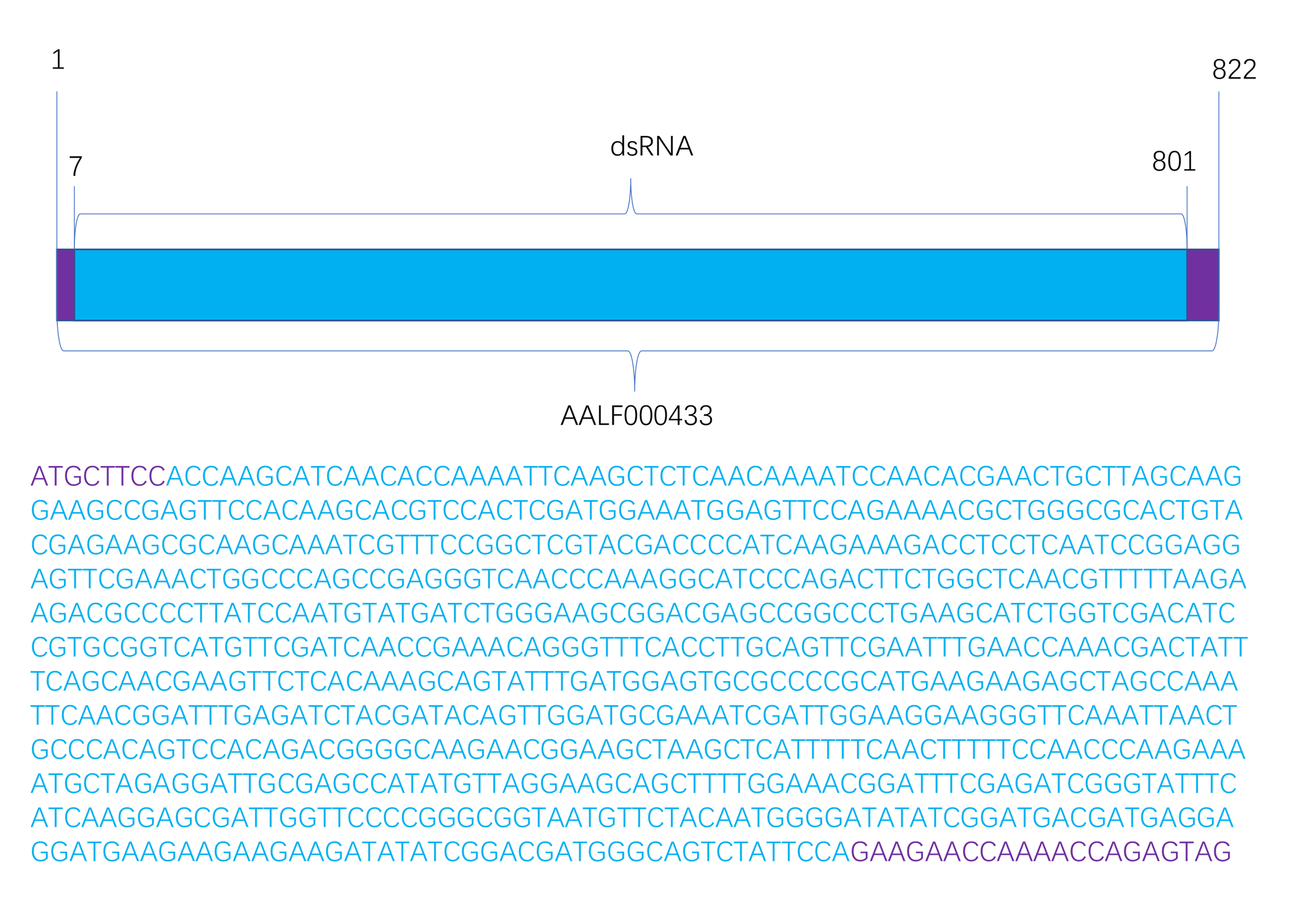

Supplement: Figure S3 — Location of dsRNA in AALF000433 and the sequence. Light Blue indicate the sequence of dsRNA. [file Image_3.jpg]
