# Supplementary material for: Differentiation of Long Non-Coding RNA and mRNA Expression Profiles in Male and Female Aedes albopictus
Source: Front Genet. 2019 Oct 14;10:975. doi: 10.3389/fgene.2019.00975 (PMC6802003; doi:10.3389/fgene.2019.00975)
Supplement: Table S4 — Primers for AALF000433 dsRNA and qRT-PCR. [file DataSheet_4.docx]

**TABLE S4 | Primers for AALF000433 dsRNA and qRT-PCR**

|  | Forward primer | Sequence (5’-3’) | Reverse primer | Sequence (5’-3’) |
| --- | --- | --- | --- | --- |
| AALF000433 Forward strand | TM00433F | GGATCCTAATACGACTCACTATAGGCCACCAAGCATCAACACCAA | M00433R | TGGAATAGACTGCCCATCGT |
| AALF000433 Reverse strand | M00433F | CCACCAAGCATCAACACCAA | TM00433R | GGATCCTAATACGACTCACTATAGGTGGAATAGACTGCCCATCGT |
| AALF000433 qRT-PCR primers | q00433F | ATCCCAGACTTCTGGCTCAA | q00433R | GGCGCACTCCATCAAATACT |
| GFP(control) | dsgfpT7F | GGATCCTAATACGACTCACTATAGGAATGGGCACAAATTTTCTGTCAGT | dsgfpT7R | GGATCCTAATACGACTCACTATAGGCCGGACTTGTATAGTTCATCCATGC |
